# Working Together

**Published:** 1866-12

**Authors:** 


### WORKING TOGETHER.

It is productive of a great deal of domestic enjoyment to have a man and wife working to the same end, having a common object in view, whether it be to save up money enough to buy a new chair, or a neighbor's adjoining farm. It is thus that they grow into the feeling that they are one, that their interests are united, and they soon begin to work into each other's hands; the wife seeks to make the husband's task easier, knowing that it enables him to do more for her, and that the common object will be the sooner attained; the husband seeing this, reciprocates, and loves the more, day by day, until they become one in aim, and feelings, and sentiments, and a love more abiding as the years grow old, is the happy result.

